# Statin Use and Major Adverse Cardiovascular Events Among Patients with Ischemic Heart Diseases: A Multi-Center Retrospective Study

**DOI:** 10.3390/jcm14030908

**Published:** 2025-01-30

**Authors:** Awatif Hafiz, Sarah Aljohani, Hussam Kutbi, Nayyara Fatani, Lama Alkhathran, Majd Alyaqub, Meshal S. Alhamed, Abdulrhaman O. Alhaqbani, Abdulrahman A. Alhadlaq, Mohammed A. Alsalman, Majed S. Al Yami, Omar A. Almohammed

**Affiliations:** 1 Department of Pharmacy Practice, Faculty of Pharmacy, King Abdulaziz University, Jeddah 22252, Saudi Arabia; whafidh@kau.edu.sa (A.H.); hkutbi1@kau.edu.sa (H.K.); nsfatani@kau.edu.sa (N.F.); 2 Department of Clinical Pharmacy, King Fahd Armed Forces Hospital, Jeddah 23311, Saudi Arabia; sarah_aljuhani@hotmail.com; 3 Department of Pharmacy Practice, College of Pharmacy, King Saud bin Abdulaziz University for Health Sciences, Riyadh 11481, Saudi Arabia; alkhathran286@gmail.com (L.A.); majdalyaqub@gmail.com (M.A.); 4 College of Medicine, King Saud University, Riyadh 11451, Saudi Arabia439100663@student.ksu.edu.sa (A.O.A.); 439106386@student.ksu.edu.sa (A.A.A.); 439100704@student.ksu.edu.sa (M.A.A.); 5 King Abdullah International Medical Research Center, Riyadh 11481, Saudi Arabia; 6 Pharmaceutical Care Department, King Abdulaziz Medical City, Riyadh 11481, Saudi Arabia; 7 Department of Clinical Pharmacy, College of Pharmacy, King Saud University, Riyadh 11451, Saudi Arabia; oalmohammed@ksu.edu.sa; 8 Pharmacoeconomics Research Unit, College of Pharmacy, King Saud University, Riyadh 11451, Saudi Arabia

**Keywords:** ischemic heart diseases, dyslipidemia, guideline, lipid management

## Abstract

**Objective**: The objective of this study was to evaluate the effects of adherence to the ACC/AHA 2018 dyslipidemia guidelines on patient management of lipid-lowering therapy in patients with ischemic heart diseases (IHD) and its correlation with major adverse cardiovascular events (MACEs), including non-fatal MI, stroke, death, hospitalization for revascularization, and peripheral arterial disease. **Methods**: A multi-center retrospective observational study was conducted in patients with IHD between January 2019 and December 2020, who were followed for two years. The primary objective was to assess statin utilization and adherence to the 2018 ACC/AHA guidelines and the associated influence on MACE outcomes. Inferential statistical analyses, including chi-square tests and the Mann–Whitney test, were conducted to assess the associations between adherence to the guidelines, MACE rates, and LDL-C goal achievement. **Results**: The study included 1011 patients with ischemic heart disease (IHD), predominantly male (78.2%), with a mean age of 59 ± 10.9 years. Non-adherent patients had higher baseline LDL-C levels (3.0 ± 1.1 mmol/L vs. 2.7 ± 1.2 mmol/L; *p* = 0.0005), while adherent patients were more likely to be on cardiovascular medications, including statins (78.4% vs. 57.4%), aspirin (74.2% vs. 56.3%), and P2Y12 inhibitors (69.5% vs. 48.4%), compared to non-adherent patients. Adherence was associated with lower non-fatal MI rates (9.3% vs. 21.1%, *p* < 0.0001) and fewer revascularizations (9.3% vs. 16.8%; *p* = 0.0024). Additionally, 49.2% of adherent patients achieved target LDL-C goals, compared to 30.5% of the non-adherent patients (*p* < 0.0001). Notably, there were no significant differences in stroke, peripheral arterial disease, or mortality rates. **Conclusions**: The achievement of target LDL-C goals and reduced MACEs was observed with adherence to the 2018 ACC/AHA dyslipidemia guidelines. However, lipid management in IHD patients remains sub-optimal, highlighting opportunities for further enhancement.

## 1. Introduction

According to the American College of Cardiology/American Heart Association (ACC/AHA) guidelines, all patients with ischemic heart disease (IHD) and other atherosclerotic cardiovascular diseases (ASCVDs)—in the absence of contraindications—should receive a high-intensity statin indefinitely. There is strong evidence supporting the use of high-intensity statins as the first-line choice for lipid management in secondary prevention to prevent the recurrence of ASCVD or cardiovascular death [1,2]. Statins are extremely effective lipid-lowering agents, which work by reducing cholesterol biosynthesis and modulating lipid metabolism in the liver [3]. In patients who have experienced an ACS episode, initiating lipid-lowering agents (especially high-intensity statins) has been shown to improve cardiovascular outcomes, regardless of previous statin exposure. Therefore, introducing a high-intensity statin (e.g., atorvastatin or rosuvastatin) as early as possible after an ACS event is recommended [4,5,6]. In addition to the ability of statins to lower circulating LDL-C, they have also been shown to be effective in stabilizing or regressing plaque through multiple mechanisms, including the reduction of necrotic lipid cores, anti-inflammatory effects, and improving endothelial function. These mechanisms give statins the ability to lower morbidity and mortality related to cardiovascular events [4,7,8]. However, low adherence to guideline recommendations for the secondary prevention of IHD is a critical issue that hinders the effectiveness of cardiovascular disease (CVD) management. Despite the well-established benefits of statin therapy in reducing the risk of IHD, studies have consistently revealed sub-optimal adherence rates among eligible patients. This non-adherence can be attributed to various factors, including patient-related factors such as a lack of awareness, concerns about side effects, and medication costs, as well as healthcare system-related barriers such as inadequate physician–patient communication and gaps in guideline implementation [9,10,11]. Improving adherence to statin therapy could significantly reduce the incidence and burden of IHD, leading to improved patient outcomes and reduced healthcare costs. Therefore, it is imperative to identify and implement targeted interventions that address the barriers to adherence and promote the appropriate use of statins, in line with evidence-based guidelines for IHD prevention.

A strong inverse relationship exists between LDL-C levels and cardiovascular outcomes, leading to the concept “the lower the LDL, the better”. This principle suggests a continuous relationship between LDL-C reduction and improved prognostic outcomes, without a specific lower limit posing risks [12]. The 2023 ESC ACS guidelines conferred values to be followed for secondary treatment; in particular, lowering LDL-C to less than 1.4 mmol/L (<55 mg/dL) and obtaining at least a 50% LDL-C reduction compared to the baseline. In another scenario, in patients experiencing a second cardiovascular event within 2 years (not necessarily of the same type as the first event), an LDL-C goal of <1.0 mmol/L (<40 mg/dL) as a treatment goal implied a greater benefit [13]. However, achieving these guideline-recommended LDL-C targets remains challenging in patients with acute coronary syndrome (ACS) [14].

This study aims to explore the relationship between statin utilization and major adverse cardiovascular events (MACEs), examining adherence to the 2018 ACC/AHA guidelines for high-intensity statin therapy in patients with IHD in Saudi Arabia. The findings of this study could inform clinical decision making, support policy development, and contribute to the overall improvement of cardiovascular disease management, potentially enhancing patient outcomes and reducing healthcare costs.

## 2. Materials and Methods

### 2.1. Study Design and Outcomes

This was a retrospective study, conducted to investigate the relationship between statin utilization according to the 2018 ACC/AHA guideline recommendations and MACEs among patients diagnosed with IHD. Adult patients (≥18 years old), who were admitted to King Abdulaziz University Hospital (KAUH) in Jeddah and King Abdulaziz Medical City (KAMC) and King Saud University Medical City (KSUMC) in Riyadh between January 2019 and December 2020, with a confirmed diagnosis of IHD at the time of enrollment, were included. The included patients were followed for two years. Patients with incomplete demographics and those prescribed high-intensity statins for other reasons than IHD were excluded. Patients were categorized into two groups: patients who were adherent to the guideline recommendations and those who were not. The data for this study were retrospectively collected from electronic medical records, including demographics, medical history, medication history, laboratory results, and MACE outcomes. The Biomedical Research Ethics Committee at all three sites approved the study, with the following reference numbers: KAUH (protocol number: 234-22), KAMC (SP22R/254/12), and KSUMC (protocol number: E-22-7285).

### 2.2. Definitions

Patients with IHD were operationally defined as patients presenting with either of the following at the time of enrollment: stable ischemic heart disease (SIHD), unstable angina (UA), non-ST elevation myocardial infarction (NSTEMI), and ST elevation myocardial infarction (STEMI). High-intensity statin was defined as receiving either rosuvastatin 20 or 40 mg or atorvastatin 40 or 80 mg. Patients who were classified as high-risk for ASCVD were those who had either multiple major ASCVDs or one major ASCVD with multiple high-risk conditions (Table 1). Adherence to the 2018 ACC/AHA guidelines was defined as meeting all three of the following criteria: (1) prescribing high-intensity or maximally tolerated statin, (2) obtaining an LDL level 4–6 weeks after statin initiation, and (3) utilization of non-statin therapy when the target LDL level was considered not to be achieved with statin alone, or if a trial with maximally tolerated statin did not achieve an LDL goal of less than 70 mg/dL. Major adverse cardiac events (MACEs) were defined as the occurrence of any of the following during the two-year follow-up period: non-fatal MI, stroke, death, hospitalization for revascularization or any cardiac cause, or peripheral arterial disease (PAD).

### 2.3. Statistical Analysis

Descriptive statistics were employed to summarize the demographic characteristics of the study population, including age, gender distribution, medical history, and medication history. The association between statin utilization and MACEs, as well as sub-group analyses based on the agent used and the doses for the adherent group, were assessed using appropriate inferential statistical analyses, including chi-square tests and the Mann–Whitney test, while adjusting for potential confounding variables.

## 3. Results

The study included a random sample of 1011 patients with IHD. The patients were predominantly male (78.2%), with an average age of 59.0 ± 10.9 years, with comparable age between the adherent and non-adherent groups (with respect to the 2018 ACC/AHA guidelines). The main comorbidities in the study population were diabetes (70.1%), hypertension (65.9%), and dyslipidemia (56.1%). These conditions were more prevalent in the non-adherent group, particularly hypertension (*p* = 0.001) and dyslipidemia (*p* = 0.0002). Additionally, heart failure was more frequent among non-adherent patients (25.8% vs. 17.1%; *p* = 0.0053). Both groups were obese on average, with a slightly higher mean body mass index (BMI) in the adherent group (30.7 ± 18.4 vs. 28.7 ± 5.1; *p* = 0.33). The medication history indicated significant differences in the use of guideline-recommended therapies between groups. Adherent patients had higher rates of using all recommended cardiovascular medications. Statin therapy was notably more frequent in the adherent group (78.4% vs. 57.4%), as were the uses of aspirin (74.2% vs. 56.3%) and P2Y12 inhibitors (69.5% vs. 48.4%). Beta-blockers and ACE inhibitors/ARBs were also more frequently prescribed to those adhering to the guidelines, underscoring a comprehensive approach to managing IHD (Table 2).

Baseline LDL-C levels were notably higher in the non-adherent group (3.0 ± 1.1 mmol/L vs. 2.7 ± 1.2 mmol/L; *p* = 0.0005), with no significant differences in other lipid profiles (Table 3). Most patients were diagnosed with their first IHD event, more frequently in the adherent group (77.8% vs. 61.1%, *p* < 0.0001). NSTEMI was the predominant initial diagnosis, affecting 50.6% of the cohort, with stent placement via percutaneous coronary intervention (PCI) being more common among non-adherent patients (41.6% vs. 30.9%; *p* = 0.0049). Following the initial IHD event, patients were predominantly classified as “high risk” (38.9%) or “very high risk” (58.7%) for future cardiovascular events, underscoring the importance of guideline adherence in this high-risk population (Table 4).

In the context of medication adherence, 809 out of the 821 adherent patients (98.5%) were on high-intensity statin therapy, primarily involving atorvastatin (40 or 80 mg) and rosuvastatin (20 or 40 mg). In contrast, only 134 of the 190 non-adherent patients (70.5%) received high-intensity statins. Although the patients had comparable rates of utilizing ezetimibe or PCSK9 inhibitors (Table 5), the use of non-statin therapies during the study period was limited to ezetimibe and PCSK9 inhibitors, with therapy escalation occurring in a subset of patients.

Clinical outcomes demonstrated significant benefits associated with adherence to guideline recommendations. Patients adhering to the guidelines showed a lower rate of non-fatal MI, with incidents occurring in 9.3% compared to 21.1% in the non-adherent group (*p* < 0.0001). Revascularization rates were also lower among adherent patients, observed at 9.3% versus 16.8% for the non-adherent group (*p* = 0.0024). Additionally, a higher percentage of adherent patients achieved target LDL-C levels at the first or second follow-up, with 49.2% meeting these targets compared to 30.5% in the non-adherent group. Moreover, the LDL-C levels were significantly lower in adherent patients after both the first and second follow-up visits. However, there were no significant differences between the groups in terms of stroke, PAD, or mortality rates (Table 6).

A sub-group analysis was performed in patients who were grouped to be adherent in order to examine the differences in outcomes with regard to the agent prescribed. When comparing rosuvastatin and atorvastatin, atorvastatin had statistically significantly lower non-fatal MI (7.1% vs. 20% *p* ≤ 0.0001), revascularization (7.7% vs. 17.3% *p* = 0.0010), and stroke (1.8% vs. 4.3%; *p* = 0.0397) levels (Table S1), and no statistical differences in outcomes were observed when the doses of these agents were further sub-analyzed (Table S2).

## 4. Discussion

In this observational study, we evaluated adherence to the 2018 ACC/AHA guideline recommendations for statin therapy in the treatment of patients with clinical ASCVD, particularly those with IHD. Our findings revealed that more than half of the study population was classified as being at very high risk, and 81.2% were adherent to guideline recommendations. More than one-third of our patients had their first episode of IHD at the time of enrollment, with 50% of these episodes being NSTEMI. Those who were adherent to the utilization patterns recommended by the guidelines had a lower incidence of non-fatal MI and hospitalization for revascularization. They were also observed to have their LDL-C at the target level during their first or second follow-up.

Maddox et al. have utilized the National Cardiovascular Data Registry’s (PINNACLE: Practice Innovation and Clinical Excellence) database to assess the effects of the 2013 ACC/AHA cholesterol guidelines on current cardiovascular practice in the United States. Their results revealed that only 50% of the patients were on statin therapy alone, while the proportion of patients on non-statin therapy was about 3%. Furthermore, statin utilization was found to be less than 80% in low- and middle-income countries [15]. We found higher rates of high-intensity statin (81%) and non-statin (24%) utilization when compared to those in the study of Maddox et al. (50% and 3%, respectively). We postulate several reasons for such findings. Unlike the study conducted by Maddox et al., in which adherence to guidelines was examined in four statin benefit groups, our study mainly focused on IHD patients, in which 50% of the cases were NSTEMI and 24% were STEMI patients, and the majority of the population was considered to be at very high risk. Thus, the prescription patterns of clinicians tend to be more aggressive for these patients. Second, all three hospitals participating in this study were teaching hospitals, and, therefore, clinicians are updated regularly. Finally, all three centers require a clinical pharmacist as part of the care team [16]. On the other hand, non-adherence to guideline recommendations was about 18%. The retrospective nature of the study limited our ability to assess the barriers to guideline adherence. Reasons for non-adherence to guidelines based on practical experience include the patient’s age (particularly age above 75), tolerability, cost, socio-economic status, and accessibility to healthcare services. All these factors warrant further exploration in subsequent studies in order to provide actionable insights and improve adherence to clinical guidelines.

It should be noted that we are still under-prescribing non-statin therapy to eligible patients. We propose several explanations for this finding. First, about 60% of our population presented with their first IHD episode, and such patients are usually not yet on a maximally tolerated statin dose. Second, the mean LDL-C at baseline was 2.8 mmol—close to the target of <2.6 mmol/L (100 mg/dL)—in which case the use of a statin alone can be expected to reduce it to target levels. Third, only 59 patients (6%) missed LDL-C monitoring at 4–6 weeks following statin initiation; thus, therapy was (in most cases) intensified as needed. It is important to note that non-statin therapies play a crucial role in helping patients to achieve target LDL-C levels, particularly in those who are unable to tolerate high-intensity statins or fail to reach target LDL-C levels despite maximal statin therapy. In our study, the overall utilization of ezetimibe was 22.7%, while PCSK9 inhibitors were used in 1.9% of patients. These therapies, when used in combination with statins, promote additional LDL-C reduction through complementary mechanisms, as supported by previous studies, in which ezetimibe has been shown to reduce LDL-C by about 30% [17], whilst PCSK9 inhibitors can cut cholesterol levels by an average of 50–60% [18]. Nevertheless, there remains a significant gap in their adoption, particularly in non-adherent patients. Potential barriers include cost considerations, limited access to PCSK9 inhibitors, and variations in the prescribing practices of clinicians.

The 2013 ACC/AHA guidelines did not specify LDL-C targets or monitoring recommendations, whereas the 2018 guidelines reintroduced these. In our study, 50% of guideline-adherent patients achieved LDL-C targets at follow-up, compared to 31% of non-adherent patients (*p* < 0.0001); meanwhile, 59 patients (6%) did not have an LDL-C value measured after statin initiation. However, this was a lower proportion of missed LDL-C lab values post-statin initiation when compared to available studies. For instance, the ACS EuroPath IV project assessed the effect of the ESC/EAS 2019 guidelines on lipid management in 2650 patients with ACS between March and June of 2022, in comparison with data collected from 2650 patients who participated in the ACS EuroPath I survey in 2018. In this study, 10% of the patients did not have lipid panel testing in 2022 [14]. Sarak et al. examined lipid testing performed in the hospital or within 90 days of discharge in patients with at least one-year survival after an ACS event between 2012 and 2018. The study included 27,979 patients, among whom 3750 patients (13.4%) did not have lipid testing [19]. It is worth noting that atherosclerotic plaque stabilization is a key mechanism through which lipid-lowering therapy (LLT) exerts its clinical benefits. High-intensity statins, in particular, play a critical role through reducing necrotic lipid cores, suppressing inflammation, and improving endothelial function, ultimately leading to more stable plaques that are less prone to rupture. Achieving LDL-C targets amplifies these effects, promoting not only the stabilization of plaque but also its regression. A recent review has emphasized that these mechanisms translate into significant reductions in major adverse cardiovascular events, including myocardial infarction and stroke [20]. In our study, patients adhering to guideline-directed LLT demonstrated higher LDL-C target attainment, which may have contributed to the observed reductions in non-fatal MI and revascularization rates. This highlights the importance of achieving LDL-C goals as a means to enhance plaque stability and improve clinical outcomes in high-risk populations.

Real-world data examining the effects of statin therapy on mortality and morbidity outcomes remain limited. In a study conducted between January 2003 and January 2011, 1528 patients who underwent PCI for ACS were followed for three months to assess all-cause mortality. About 60% of the patients were on high-intensity statins, while 40% were either on a low-dose statin or not on statins at all. A statistically significant reduction in all-cause mortality during the 3-month follow-up was observed in those receiving high-intensity statins. All-cause mortality occurred in 8 patients (0.9%) receiving high-intensity statin therapy and 21 patients (3.5%) taking low-intensity statins or no statin therapy at discharge (hazard ratio 0.244, 95% confidence interval [CI] 0.108–0.551; *p* = 0.001) [21]. Although not statistically significant, our study observed a numerically lower incidence of mortality due to cardiovascular disease (1.7% vs. 3.7%) or death from any cause (1.8% vs. 2.1%) among patients who were adherent to guideline recommendations, mostly receiving high-intensity statins (98.5% of these patients). However, this numerically lower incidence can be explained by the statistically significant difference in utilization rates of recommended therapies for secondary prevention of ACS, such as aspirin, P2Y_12_ inhibitors, beta-blockers, ACEI/ARBs, and spironolactone.

To shed light on morbidity-related outcomes such as non-fatal MI, hospitalization, and/or revascularization, Timothy et al. conducted a meta-analysis of RCTs or systematic reviews on coronary heart disease to determine the effectiveness of statins. In particular, RCTs or systematic reviews published between January 1966 and December 2002 were included, for a total of 25 studies enrolling 69,511 individuals. Statin therapy reduced non-fatal MI by 25% (relative risk 0.75; 95%CI, 0.71–0.79) [22]. Our study revealed a statistically significant lower incidence of non-fatal MI between the group who were adherent to the guidelines and those who were not (9.3% vs. 21.1%; *p* < 0.0001). Additionally, hospitalization for revascularization was also statistically significant between the two groups (9.3% vs. 16.8%; *p* < 0.0024). Notably, it was observed that when a lower LDL-C level was achieved, a greater benefit in terms of ASCVD reduction was obtained. As mentioned above, about 50% of the patients in the group who were adherent to the guidelines had their LDL-C at target (<70 mg/dL), compared to 31% of those patients who were not in alignment with guidelines.

Although our study was a multi-center study, it had some limitations. The retrospective nature of the study might introduce some documentation bias due to the complexity of the chart review process. This might have also led to the difficulty of assessing adverse events that highly impact statin adherence. Compared to other real-world data studies, we had a small sample size. In addition, a reduced amount of data collection occurred during the COVID-19 pandemic period, which may have affected access to and availability of laboratory testing. Furthermore, in the middle of the study period, the 2022 ACC Expert Consensus Report further reduced the threshold for consideration of non-statin therapy to 55 mg/dL for patients with clinical ASCVD who are at very high risk. However, we doubt that this alteration impacted our results, as it was published in November of 2022, while our patients were followed to the end of 2022 only.

## 5. Conclusions

In conclusion, this study is among the first to evaluate adherence to the 2018 ACC/AHA guidelines for lipid management in a real-world, multi-center setting within a Saudi Arabian population. Our findings highlighted the substantial benefits of adherence, including improved LDL-C goal attainment and reductions in non-fatal MI and revascularization rates. The novelty of this study lies in its focus on a diverse, multi-racial population living in Saudi Arabia, where limited data regarding the applicability of international guidelines are available at present. By demonstrating that the benefits of guideline-directed therapies extend to this population, we provide a foundation for assessing current prescribing practices and identifying actionable strategies to optimize care and outcomes in this unique context. To further enhance adherence, we conclude that including a pharmacist in the care team, who can update the team via teaching once relevant guidelines are updated, could potentially help to bring prescribing patterns in line with the recommendations of such guidelines. Several postulated theories should be further examined for their capacity to enhance LDL testing and further target level achievement, including reminding patients about their upcoming lab tests, as well as monitoring the tolerability and utilization of non-statin therapy when effective levels of statins are deemed intolerable.

## Figures and Tables

**Table 1:** Very high risk for future ASCVD events [1].

Major ASCVD Events	High-Risk Conditions
Recent ACS (within 12 month) History of MI (other than the recent ACS) History of ischemic stroke Symptomatic PAD (history of claudication with ABI < 0.85 or previous revascularization or amputation)	Age ≥ 65 y Heterozygous familial hypercholesterolemia History of prior CABG or PCI outside of the major ASCVD event(s) Diabetes mellitus Hypertension Chronic kidney disease (eGFR 15–59 mL/min/1.73 m^2^) Smoking Persistently elevated LDL-C (LDL-C ≥ 100 mg/dL) despite maximally tolerated statin therapy and ezetimibe History of congestive heart failure

ACS: acute coronary syndrome, MI: myocardial infarction, PAD: peripheral arterial disease, ABI: ankle brachial index, CABG: coronary artery bypass grafting, PCI: percutaneous coronary intervention, eGFR: glomerular filtration rate, LDL-C: low-density lipoprotein.

**Table 2:** Baseline demographics and clinical characteristics of patients based on adherence to the guideline’s recommendations.

Characteristics	All Patients N = 1011	Adherence to Guideline Recommendations		*p*-Value ^†^
		No N = 190 (18.8%)	Yes N = 821 (81.2%)	
Age	59.0 ± 10.9	59.5 ± 11.0	58.9 ± 10.9	0.4206
Male	791 (78.2)	137 (72.1)	654 (79.7)	**0.0230**
Body mass index (BMI)	30.3 ± 16.7	28.7 ± 5.1	30.7 ± 18.4	0.3328
Active smokers	306 (30.3)	53 (27.9)	253 (30.8)	0.5783
Comorbidities				
Diabetes mellitus	709 (70.1)	141 (74.2)	568 (69.2)	0.3909
Hypertension	666 (65.9)	146 (76.8)	520 (63.3)	**0.0013**
Dyslipidemia	567 (56.1)	132 (69.5)	435 (53.0)	**0.0002**
Heart failure	189 (18.6)	49 (25.8)	140 (17.1)	**0.0053**
HFrEF	161 (15.9)	44 (23.2)	117 (14.3)	**0.0098**
HFpEF	28 (2.8)	5 (2.6)	23 (2.8)	0.9458
Chronic kidney disease	84 (8.3)	23 (12.1)	61 (7.4)	0.0713
Ischemic stroke or TIA	46 (4.5)	7 (3.7)	39 (4.8)	0.4341
Hemorrhagic stroke	5 (0.5)	0 (0.0)	5 (0.6)	0.4502
Atrial fibrillation	37 (3.7)	10 (5.3)	27 (3.3)	0.3405
Peripheral arterial disease	16 (1.6)	1 (0.5)	15 (1.8)	0.4319
History of carotid stenosis	6 (0.6)	0 (0.0)	6 (0.7)	0.3108
Medication History				
Lipid-lowering agents	753 (74.5)	109 (57.4)	644 (78.4)	**<0.0001**
Aspirin	716 (70.8)	107 (56.3)	609 (74.2)	**<0.0001**
P2Y12 inhibitors	663 (65.6)	92 (48.4)	571 (69.5)	**<0.0001**
Beta Blockers	663 (65.6)	92 (48.4)	571 (69.5)	**<0.0001**
ACEI/ARB	563 (55.7)	80 (42.1)	483 (58.8)	**<0.0001**
Spironolactone	83 (8.2)	6 (3.2)	77 (9.4)	**<0.0001**

Numbers are presented as mean ± SD or frequency (%). ^†^ *p*-values are from the Mann–Whitney test for continuous not normally distributed data or chi-square test for categorical data; values in bold are statistically significant. Abbreviations: SD: standard deviation; BMI: body mass index; HFrEF: heart failure with reduced ejection fraction; HFpEF: heart failure with preserved ejection fraction; TIA: transient ischemic attack; ACEI: angiotensin-converting enzyme inhibitors; ARB: angiotensin receptor blocker.

**Table 3:** Laboratory values at baseline based on adherence to the guideline’s recommendations.

Laboratory Test	All Patients	Adherence to Guideline Recommendations		*p*-Value ^†^
		No	Yes	
**HbA1C, %**	8.0 ± 2.2	8.3 ± 2.3	7.9 ± 2.2	**0.0481**
**Total cholesterol, mmol/L**	4.4 ± 1.3	4.6 ± 1.3	4.4 ± 1.3	0.0747
**LDL-C, mmol/L**	2.8 ± 1.2	3.0 ± 1.1	2.7 ± 1.2	**0.0005**
**HDL-C, mmol/L**	1.0 ± 0.3	1.0 ± 0.2	1.0 ± 0.3	0.3386
**Triglyceride, mmol/L**	1.8 ± 1.1	1.9 ± 1.3	1.8 ± 1.1	0.4192
**Serum creatinine, mg/dL**	1.2 ± 0.9	1.1 ± 0.7	1.2 ± 1.0	0.3629
**Creatinine clearance, mL/min**	90.8 ± 38.9	88.0 ± 36.2	91.4 ± 39.5	0.4051

Numbers are presented as mean ±SD. ^†^ *p*-values are from the Mann–Whitney test for continuous not normally distributed data; values in bold are statistically significant. Abbreviations: SD: standard deviation.

**Table 4:** Classification of the index IHD event based on adherence to the guideline’s recommendations.

Baseline Incident and Procedure	All Patients N = 1011	Adherence to Guideline Recommendations		*p*-Value ^†^
		No N = 190 (18.8%)	Yes N = 821 (81.2%)	
Classification of the new IHD				**0.0172**
NSTEMI	512 (50.6)	105 (55.3%)	407 (49.6%)	
STEMI	241 (23.8)	28 (14.7%)	213 (25.9%)	
UA	108 (10.7)	26 (13.7%)	82 (10.0%)	
Stable IHD	15 (1.5)	2 (1.1%)	13 (1.6%)	
Number of diseased vessels				0.2038
One	306 (30.3)	46 (24.2)	260 (31.7)	
Two	181 (17.9)	37 (19.5)	144 (17.5)	
Three	208 (20.6)	37 (19.5)	171 (20.8)	
Four	91 (9.0)	21 (11.1)	70 (8.5)	
New or recurrent event				**<0.0001**
New	755 (74.7)	116 (61.1)	639 (77.8)	
Recurrent	230 (22.7)	65 (34.2)	165 (20.1)	**0.0215**
2nd	177 (17.5)	44 (23.2)	133 (16.2)	
3rd	36 (3.6)	14 (7.4)	22 (2.7)	
4th	12 (1.2)	3 (1.6)	9 (1.1)	
5th or more	5 (0.5)	4 (2.1)	1 (0.1)	
Not documented	26 (2.6)	9 (4.7)	17 (2.1)	
Procedure performed				
Stent PCI	333 (32.9)	79 (41.6)	254 (30.9)	**0.0049**
CABG	208 (20.6)	36 (18.9)	172 (21.0)	0.5383
Unspecified PCI	199 (19.7)	15 (7.9)	184 (22.4)	**<0.0001**
Medical therapy only	167 (16.5)	44 (23.2)	123 (15.0)	**0.0062**
Balloon PCI	47 (4.6)	7 (3.7)	40 (4.9)	0.4834
Not documented	94 (9.3)	17 (8.9)	77 (9.4)	0.8536
Risk category (after event)				0.2504
High risk	393 (38.9)	84 (44.2)	309 (37.6)	
Very high risk	593 (58.7)	102 (53.7)	491 (59.8)	

Numbers are presented as frequency (%). ^†^ *p*-values are from the chi-square test; values in bold are statistically significant. Abbreviations: IHD: ischemic heart disease; STEMI: ST-elevation myocardial infarction; NSTEMI: non-ST-elevation myocardial infarction; UA: unstable angina; PCI: percutaneous coronary intervention; CABG: coronary artery bypass grafting.

**Table 5:** Lipid-lowering agents used after the index event and its distribution based on adherence to the guideline’s recommendations.

Outcomes	All Patients N = 1011	Adherence to Guideline Recommendations		*p*-Value ^†^
		No N = 190 (18.8%)	Yes N = 821 (81.2%)	
Statin agent used				**<0.0001**
Atorvastatin	780 (77.1)	100 (52.6)	680 (82.7)	
10 mg	4 (0.4)	3 (1.6)	1 (0.1)	
20 mg	29 (2.9)	27 (14.2)	2 (0.2)	
40 mg	417 (41.2)	44 (23.2)	373 (45.4)	
80 mg	330 (32.6)	26 (13.7)	304 (37.0)	
Rosuvastatin	220 (21.8)	80 (42.1)	140 (17.1)	
10 mg	24 (2.4)	16 (8.4)	8 (1.0)	
20 mg	158 (15.6)	52 (27.4)	106 (12.9)	
40 mg	38 (3.8)	12 (6.3)	26 (3.2)	
Simvastatin	11 (1.1)	10 (5.3)	1 (0.1)	
10 mg	7 (0.7)	7 (3.7)	0 (0.0)	
20 mg	4 (0.4)	3 (1.6)	1 (0.1)	
Additional drug used				
Ezetimibe	229 (22.7)	37 (19.5)	192 (23.4)	0.5095
PCSK9 inhibitors	19 (1.9)	2 (1.1)	17 (2.1)	0.6470

Numbers are presented as frequency (%). ^†^ *p*-values are from the chi-square test; values in bold are statistically significant. Abbreviation: PCSK9: Proprotein convertase subtilisin/kexin type 9.

**Table 6:** Patients’ outcomes based on adherence to the guideline’s recommendations.

Outcomes	All Patients N = 1011	Adherence to Guideline Recommendations		*p*-Value ^†^
		No N = 190 (18.8%)	Yes N = 821 (81.2%)	
Patient was at LDL-C goal at 1st or 2nd follow-up	462 (45.7)	58 (30.5)	404 (49.2)	**<0.0001**
LDL-C at first follow-up	2.1 ± 1.0	2.4 ± 1.1	2.0 ± 1.0	**0.0009**
LDL-C at second follow-up	1.9 ± 0.9	2.2 ± 0.8	1.9 ± 0.9	**0.0257**
Non-fatal MI	116 (11.5)	40 (21.1)	76 (9.3)	**<0.0001**
Revascularization	108 (10.7)	32 (16.8)	76 (9.3)	**0.0024**
Stroke	25 (2.5)	7 (3.7)	18 (2.2)	0.2328
Peripheral arterial disease	2 (0.2)	1 (0.5)	1 (0.1)	0.2621
Death from CVD	21 (2.1)	7 (3.7)	14 (1.7)	0.0848
Death due to any cause	19 (1.9)	4 (2.1)	15 (1.8)	0.7991

Numbers are presented as frequency (%). ^†^ *p*-values are from the chi-square test; values in bold are statistically significant. Abbreviations: MI: myocardial infarction; CVD: cardiovascular disease.

## Data Availability

The datasets used and/or analyzed during the current study are available from the corresponding author upon reasonable request.

## Supplementary Materials

The following supporting information can be downloaded at: https://www.mdpi.com/article/10.3390/jcm14030908/s1, Table S1: Adherant patients’ outcomes based on agents used; Table S2: Adherant patients’ outcomes based on agents’ doses used.

## References

[1] Grundy S.M., Stone N.J., Bailey A.L., Beam C., Birtcher K.K., Blumenthal R.S., Braun L.T., de Ferranti S., Faiella-Tommasino J., Forman D.E. (2019). 2018 AHA/ACC/AACVPR/AAPA/ABC/ACPM/ADA/AGS/APhA/ASPC/NLA/PCNA Guideline on the Management of Blood Cholesterol: Executive Summary: A Report of the American College of Cardiology/American Heart Association Task Force on Clinical Practice Guidelines. J. Am. Coll. Cardiol..

[2] Smith S.C., Benjamin E.J., Bonow R.O., Braun L.T., Creager M.A., Franklin B.A., Gibbons R.J., Grundy S.M., Hiratzka L.F., Jones D.W. (2011). AHA/ACCF Secondary Prevention and Risk Reduction Therapy for Patients with Coronary and other Atherosclerotic Vascular Disease: 2011 update: A guideline from the American Heart Association and American College of Cardiology Foundation. Circulation.

[3] Stancu C., Sima A. (2001). Statins: Mechanism of action and effects. J. Cell Mol. Med..

[4] Rosa G.M., Carbone F., Parodi A., Massimelli E.A., Brunelli C., Mach F., Vuilleumier N., Montecucco F. (2014). Update on the efficacy of statin treatment in acute coronary syndromes. Eur. J. Clin. Investig..

[5] Ference B.A., Ginsberg H.N., Graham I., Ray K.K., Packard C.J., Bruckert E., Hegele R.A., Krauss R.M., Raal F.J., Schunkert H. (2017). Low-density lipoproteins cause atherosclerotic cardiovascular disease. 1. Evidence from genetic, epidemiologic, and clinical studies. A consensus statement from the European Atherosclerosis Society Consensus Panel. Eur. Heart J..

[6] Navarese E.P., Kowalewski M., Andreotti F., van Wely M., Camaro C., Kolodziejczak M., Gorny B., Wirianta J., Kubica J., Kelm M. (2014). Meta-analysis of time-related benefits of statin therapy in patients with acute coronary syndrome undergoing percutaneous coronary intervention. Am. J. Cardiol..

[7] Baigent C., Keech A., Kearney P.M., Blackwell L., Buck G., Pollicino C., Kirby A., Sourjina T., Peto R., Collins R. (2005). Efficacy and safety of cholesterol-lowering treatment: Prospective meta-analysis of data from 90,056 participants in 14 randomised trials of statins. Lancet.

[8] Ward N.C., Watts G.F., Eckel R.H. (2019). Statin Toxicity. Circ. Res..

[9] Arnold S.V., Spertus J.A., Tang F., Krumholz H.M., Borden W.B., Farmer S.A., Ting H.H., Chan P.S. (2011). Statin use in outpatients with obstructive coronary artery disease. Circulation.

[10] Cassagnol M., Hai O., Sherali S.A., D’Angelo K., Bass D., Zeltser R., Makaryus A.N. (2020). Impact of cardiologist intervention on guideline-directed use of statin therapy. World J. Cardiol..

[11] Schoen M.W., Salas J., Scherrer J.F., Buckhold F.R. (2015). Cholesterol treatment and changes in guidelines in an academic medical practice. Am. J. Med..

[12] Pedro-Botet J., Pintó X. (2019). Colesterol LDL, cuanto más bajo mejor. Clínica Investig. Arterioscler..

[13] Byrne R.A., Rossello X., Coughlan J.J., Barbato E., Berry C., Chieffo A., Claeys M.J., Dan G.A., Dweck M.R., Galbraith M. (2023). 2023 ESC Guidelines for the management of acute coronary syndromes. Eur. Heart J..

[14] Laufs U., Catapano A.L., de Caterina R., Schiele F., Sionis A., Zaman A., Jukema J.W. (2023). The effect of the 2019 ESC/EAS dyslipidaemia guidelines on low-density lipoprotein cholesterol goal achievement in patients with acute coronary syndromes: The ACS EuroPath IV project. Vasc. Pharmacol..

[15] Maddox T.M., Borden W.B., Tang F., Virani S.S., Oetgen W.J., Mullen J.B., Chan P.S., Casale P.N., Douglas P.S., Masoudi F.A. (2014). Implications of the 2013 ACC/AHA cholesterol guidelines for adults in contemporary cardiovascular practice: Insights from the NCDR PINNACLE registry. J. Am. Coll. Cardiol..

[16] Cornelison P., Marrs J.C., Anderson S.L. (2021). Clinical Pharmacist Outreach to Increase Statin Use for Patients with Cardiovascular Disease in a Safety-Net Healthcare System. Am. Health Drug Benefits.

[17] Cannon C.P., Blazing M.A., Giugliano R.P., McCagg A., White J.A., Theroux P., Darius H., Lewis B.S., Ophuis T.O., Jukema J.W. (2015). Ezetimibe Added to Statin Therapy after Acute Coronary Syndromes. N. Engl. J. Med..

[18] Sabatine M.S., Giugliano R.P., Keech A.C., Honarpour N., Wiviott S.D., Murphy S.A., Kuder J.F., Wang H., Liu T., Wasserman S.M. (2017). Evolocumab and Clinical Outcomes in Patients with Cardiovascular Disease. N. Engl. J. Med..

[19] Sarak B., Savu A., Kaul P., McAlister F.A., Welsh R.C., Yan A.T., Goodman S.G. (2021). Lipid Testing, Lipid-Modifying Therapy, and PCSK9 (Proprotein Convertase Subtilisin-Kexin Type 9) Inhibitor Eligibility in 27 979 Patients With Incident Acute Coronary Syndrome. Circ. Cardiovasc. Qual. Outcomes.

[20] Cesaro A., Acerbo V., Indolfi C., Filardi P.P., Calabrò P. (2024). The Clinical Relevance of the Reversal of Coronary Atherosclerotic Plaque. Eur. J. Intern. Med..

[21] Tentzeris I., Rohla M., Jarai R., Farhan S., Freynhofer M.K., Unger G., Nürnberg M., Geppert A., Wessely E., Wojta J. (2014). Influence of High-Dose Highly Efficient Statins on Short-Term Mortality in Patients Undergoing Percutaneous Coronary Intervention With Stenting for Acute Coronary Syndromes. Am. J. Cardiol..

[22] Wilt T.J., Bloomfield H.E., MacDonald R., Nelson D., Rutks I., Ho M., Larsen G., McCall A., Pineros S., Sales A. (2004). Effectiveness of statin therapy in adults with coronary heart disease. Arch. Intern. Med..

