# Supplementary material for: Statin Use and Major Adverse Cardiovascular Events Among Patients with Ischemic Heart Diseases: A Multi-Center Retrospective Study
Source: J Clin Med. 2025 Jan 30;14(3):908. doi: 10.3390/jcm14030908 (PMC11818335; doi:10.3390/jcm14030908)
Supplement: Supplementary file 1 [file jcm-14-00908-s001.zip › jcm-3414661-supplementary.pdf]

Table S1. Adherant patients' outcomes based on agents used*

Outcomes	All Patients N = 820	Agent		***P*-value**†
		**Atorvastatin** N = 680 (82.9%)	**Rosuvastatin** N = 140 (17.1%)	
**Patient was at LDL-C goal at 1st or 2nd follow-up**	404 (49.3)	328 (48.2)	76 (54.3)	0.1922
**Non-fatal MI**	76 (9.3)	48 (7.1)	28 (20.0)	**<0.0001**
**Revascularization**	76 (9.3)	52 (7.7)	24 (17.3)	**0.0010**
**Stroke**	18 (2.2)	12 (1.8)	6 (4.3)	**0.0397**
**Peripheral arterial disease**	1 (0.1)	1 (0.2)	0 (0.0)	0.7209
**Death from CVD**	14 (1.7)	11 (1.6)	3 (2.1)	0.6376
**Death due to any cause**	15 (1.8)	11 (1.6)	4 (2.9)	0.2893

Numbers are presented as frequency (%)

* Only one patient received simvastatin in this group and was excluded from this analysis.

†*p*-values are from the Chi-Square test; values in bold are statistically significant. Abbreviations: MI: myocardial infarction; CVD: cardiovascular disease.

Table S2. Adherant patients' outcomes based on agents' doses used*

Outcomes	**Atorvastatin** N = 680 (82.9%)					**Rosuvastatin** N = 140 (17.1%)			
	10 mg (n=1)	20 mg (n=2)	40 mg (n=373)	80 mg (n=303)	*P*-value†	10 mg (n=8)	20 mg (n=106)	40 mg (n=26)	*P*-value†
**Patient was at LDL-C goal at 1st or 2nd follow-up**	1 (100.0)	2 (100.0)	177 (47.5)	148 (44.6)	0.3402	7 (87.5)	53 (50.0)	16 (61.5)	0.0866
**Non-fatal MI**	0 (0.0)	0 (0.0)	35 (9.4)	13 (4.3)	0.2065	1 (12.5)	22 (20.75)	5 (19.2)	0.8485
**Revascularization**	0 (0.0)	0 (0.0)	38 (10.2)	14 (4.6)	0.2528	0 (0.0)	17 (16.2)	7 (26.9)	0.0614
**Stroke**	0 (0.0)	0 (0.0)	6 (1.6)	6 (2.0)	0.9996	1 (12.5)	3 (2.8)	2 (7.7)	0.2727
**Peripheral arterial disease**	0 (0.0)	0 (0.0)	1 (2.3)	0 (0.0)	0.9743	0 (0.0)	0 (0.0)	0 (0.0)	1.0000
**Death from CVD**	0 (0.0)	0 (0.0)	7 (1.9)	4 (1.3)	0.9715	1 (12.5)	0 (0.0)	2 (7.7)	0.0014
**Death due to any cause**	0 (0.0)	0 (0.0)	8 (2.1)	8 (2.6)	0.9465	0 (0.0)	3 (2.8)	1 (3.9)	0.5668

Numbers are presented as frequency (%)

* Only one patient received simvastatin in this group and was excluded from this analysis.

†*p*-values are from the Chi-Square test; values in bold are statistically significant. Abbreviations: MI: myocardial infarction; CVD: cardiovascular disease.
